# Tracing psychological resources and mental health: the role of sense of coherence, resilience and self-efficacy in young adulthood

**DOI:** 10.3389/frcha.2026.1759711

**Published:** 2026-04-30

**Authors:** Julia Franziska Baschab, Sena Aktürk, Hannah Honecker-Gebauer, Eva Moehler, Justine Hussong

**Affiliations:** 1 Child and Adolescence Psychiatry, Saarland University, Homburg, Germany; 2 Child and Adolescent Psychiatry, Saarland University Medical Center, Homburg, Germany

**Keywords:** locus of control (LOC), longitudinal study, mental health, optimism, psychological resources, resilience, self-efficacy, sense of coherence (SOC)

## Abstract

**Background:**

Psychological resources such as resilience, sense of coherence (SOC), optimism, self-efficacy, and locus of control are central to adaptive functioning and mental health. However, their developmental antecedents and interrelations remain insufficiently understood.

**Methods:**

Using longitudinal data from a population-based cohort, this study examined a) associations between psychological resources and psychopathology in young adulthood (18–19 years), and b) early predictors of these resources, including psychopathology, social competences, maternal psychopathology, and bonding. An exploratory factor analysis was conducted to test the structure of psychological resources.

**Results:**

Higher levels of resilience, SOC, optimism, and self-efficacy were associated with lower psychopathology symptoms in young adulthood, even after controlling for early risk factors. SOC emerged as the strongest unique predictor, indicating a central integrative role. Exploratory factor analysis supported a dominant general psychological resource dimension. Psychopathology predicted lower psychological resources, whereas maternal factors showed limited and inconsistent direct effects.

**Conclusions:**

Psychological resources are closely intertwined with mental health in young adulthood, with SOC appearing as a particularly central component. These findings underscore the relevance of psychological resources for understanding individual differences in mental health and suggest their potential value as targets for future longitudinal and intervention-based research.

## 1 Introduction

The early years of life lay the groundwork for emotional and social development across the lifespan. In children, socioemotional competences, particularly emotion regulation and coregulation with caregivers, emerge through rich interactions in the family system shaping how emotions are perceived, expressed, and regulated (1, 2). Emotion regulation, the ability to modulate one's own affective states, plays a central role and is strongly influenced by parental sensitivity and caregiving quality (3–5).

Early maternal bonding has been identified as a key mechanism shaping long-term socioemotional functioning. Bonding refers to the parent's emotional connection to the child, distinct from attachment, which reflects the child's emotional tie to the caregiver (6, 7). Sensitive maternal bonding in the early postnatal period fosters secure attachment relationships, emotional attunement, and long-term social-emotional competence (7, 8). Conversely, bonding impairments have been linked to affective dysregulation, heightened psychopathology risk, social competences, and difficulties in forming close relationships throughout life (9–12).

Salutogenetic resources are grounded in Antonovsky's salutogenetic framework, which emphasizes factors that promote health and well-being rather than merely protect against disease (13). Within this framework, sense of coherence (SOC), resilience, self-efficacy, and optimism represent central protective mechanisms that help individuals maintain mental health despite adversity (14–17). SOC reflects the perception of life as meaningful, manageable, and comprehensible, integrating beliefs about competence, control, and positive expectations (13). Resilience refers to the capacity to recover from negative events, such as adversity or significant stress, enabling individuals to maintain adaptive functioning under challenging conditions (18, 19). Self-efficacy denotes confidence in one's own abilities to achieve goals and exert control over outcomes, supporting proactive coping and problem-solving (20). Locus of control describes the extent to which individuals perceive outcomes as contingent on their own actions vs. external factors, influencing motivation and adaptive behavior (21). Optimism reflects the general expectations that positive outcomes will occur, promoting proactive coping, perseverance, and positive adjustment in the face of adversity (22). Resilience, self-efficacy, locus of control, and optimism complement SOC by enabling adaptive coping, recovery from stress, confidence in personal capacities and personal agency (21, 23–27). These resources often co-occur and interact, forming a cohesive psychological system that protects against psychopathology and promotes adaptive development, particularly during adolescence and early adulthood, when identity formation and environmental demands intensify (19, 28).

From a developmental psychopathology perspective, early risk and protective factors interact across time to shape adaption (29). Longitudinal evidence indicates that early maternal mental health, bonding quality, and caregiving experiences exert long-term influences on socioemotional development and psychological resources, moderated by child temperament and gender (12, 30). Yet, little is known about how these early influences translate into the development of resilience, SOC, optimism, self-efficacy, and locus of control in young adulthood, or how these resources relate to subsequent psychopathological symptoms.

### 1.1 The present study (T7)

The present study examines psychosocial resources and psychopathological symptoms in young adulthood and their early maternal and child-related predictors in a longitudinal cohort followed from birth. Resilience, sense of coherence, optimism, self-efficacy, and locus of control are conceptualized as interrelated psychological resources that jointly support adaptive functioning and mental health. Modeling them together allows us to examine both shared and unique contributions and to test for a general psychological resource factor underlying these constructs, providing a more comprehensive understanding of individual differences in young adult mental health.

Findings from the previous waves (timepoints T1–T6) (12, 30–34) of this study illustrate the enduring impact of early maternal mental health, bonding quality, and maternal psychopathology on child socioemotional, behavioral, and personality development. Yet, to date no study has investigated whether early maternal and child factors predict resilience, sense of coherence, optimism, locus of control, and self-efficacy in early adulthood, or how these resources in turn, relate to psychopathological symptoms. Addressing this gap may clarify the long-term pathways from early influences to psychosocial outcomes across two decades of life. Building on this longitudinal work, the present study (T7, 18–19 years) examines how early maternal mental health, bonding quality, maternal psychopathology, and child behavioral dispositions jointly predict psychosocial outcomes in young adulthood. Specifically, the current study seeks to answer two central questions:

1. Do resilience, sense of coherence (SOC), optimism, locus of control, and self-efficacy predict psychopathological symptoms in young adulthood (18–19 years)?
2. Do perinatal, postnatal, early childhood, and child behavioral factors predict resilience, sense of coherence (SOC), optimism, locus of control, and self-efficacy in young adulthood (18–19 years)?

Based on these research questions, the following hypotheses are proposed:

H1: Associations with mental health

In young adulthood (T7; 18–19 years), lower levels of psychopathological symptoms will be associated with a:

1. H1a: higher resilience at T7.
2. H1b: stronger sense of coherence (SOC) at T7.
3. H1c: higher optimism at T7.
4. H1d: higher internal locus of control at T7.
5. H1e: higher self-efficacy at T7.

H2: Early predictors of psychosocial resources (exploratory)

Early child and adolescent variables (sex, T1; social competences, T5; psychopathology, T6) and maternal variables (psychopathology, mean T1–T5; bonding quality, mean T1–T4; and education, T1), may predict:

1. H2a: resilience at T7 (18–19 years).
2. H2b: sense of coherence (SOC) at T7 (18–19 years).
3. H2c: optimism at T7 (18–19 years).
4. H2d: locus of control at T7 (18–19 years).
5. H2e: self-efficacy at T7 (18–19 years).

## 2 Method

This study was pre-registered before data analyses on AsPredicted (https://aspredicted.org/2ft8ma.pdf), including all planned analyses at T7, to ensure transparency and reproducibility of the research questions, hypotheses, and planned analyses.

### 2.1 Participants

Participants were drawn from a neonatal cohort recruited as part of two German Research Foundation projects MO978/1–1 and MO978/1–2 (31–33). Inclusion criteria were full-term singleton births (≥37 weeks of gestation), birth weight >2,500 g, and Apgar scores >7. Mothers required sufficient German language proficiency, and pregnancies with substantial risk factors (e.g., maternal drug use, alcohol misuse, >5 cigarettes/day) were excluded. Written informed consent was obtained from all participants, and participation was voluntary at all stages, with the option to withdraw at any time without reason.

Between April 2002 and October 2004, 342 mothers were approached at four major obstetric units serving a mixed urban and rural population. Of these, 114 consented, and 101 mother-infant dyads ultimately completed the baseline assessment (T1). Mothers had a mean age of *M* = 33.3 years (*SD* = 4.13, range = 25.45), 81% held a college degree, 18% a high school degree, and 44.6% of infants were female.

Across seven assessment waves (T1–T7), retention decreased from 100% at T1 to 50.5% at T7 (see Table 1). Reasons for attrition included relocation, time constraints, maternal death, and unspecified factors. The final T7 sample included 51 young adults (*M*_age_ = 18.65 years, *SD*_age_ = 0.48 years; 51% female). No *a priori* power or sample size calculation was performed, as the study was designed as a longitudinal observational cohort.

**Table 1:** Participant flow across assessment points.

Timepoint	Child's age	N included	Retention (%)
T1	2 weeks	101	100.0%
T2	6 weeks	101	100.0%
T3	4 months	101	100.0%
T4	14 months	98	97.0%
T5	5.5 years	88	87.1%
T6	14 years	76	75.2%
T7	18–19 years	51	50.5%

T7 retention is calculated relative to baseline (T1 = 101 dyads).

### 2.2 Prior longitudinal studies of T1–T6

Earlier analyses from previous measurement waves (T1–T6) of this cohort have demonstrated consistent associations between early maternal mental health, bonding quality, and later socioemotional as well as personality development (12, 30–34). Higher maternal depressive symptoms in infancy (T1–T4) were associated with impaired bonding and greater infant behavioral inhibition (31–33). Early bonding quality predicted children's social competence at age 5.5 years (12), and bonding impairment in the early postpartum period was related to adolescent personality dysfunction, moderated by child temperament and sex (30). Moreover, maternal psychopathology across the first 5.5 years further contributed to impaired identity development, as children of mothers with higher symptom levels showed greater difficulties in forming a coherent identity (34).

Assessments included maternal psychopathology [SCL-90-R; (35, 36)], maternal personality [German version NEO-FFI; (37, 38)], prenatal emotional stress [PESI; (32)], life events and stressors in pregnancy [LEBI; (39)], medical pregnancy data [Steinhausen score; (36)], postpartum depression [EPDS; (40, 41)], maternal bonding [PBQ; (42)], infant reactivity at 4 months (43), behavioral inhibition at 14 months (44, 45), social competences [SOCOMP; (46)], temperament and character [JTCI 3-6R; (47)], infant behavior [IBQ (48);], behavioral inhibition (44), borderline personality features [CI-BPD; (49)] and psychopathology [BSI-18; (50)], personality functioning [LPFS-BF 2.0; (47, 48)].

Further details on these measures are provided in (12, 30, 32–34, 45, 51).

### 2.3 Measures of T7 outcomes

*Resilience* was measured with the 13-item Resilience Scale (RS-13 [RS-13; (52)] on a 7-point scale. Higher scores indicate greater resilience. Cronbach's α in this sample was 0.86.

*Sense of coherence* was assessed with the 9-item Sense of Coherence Scale-Short-form [SOC-9; (53)], covering on a 7-point scale comprehensibility, manageability, and meaningfulness. Higher scores indicate stronger SOC. Cronbach's α in this sample was 0.88.

*Self-Efficacy* was assessed with the General Self-Efficacy Scale [ASKU; (54)], 10 items on a 4-point scale, with higher scores indicating greater self-efficacy.

*Optimism* was measured with the Life Orientation Test—Revised [SOP2; (55)], 10 items (6 scored, 4 fillers) on a 5-point scale, with higher scores indicating greater optimism.

*Locus of Control* was assessed with the Internal-External Locus of Control Scale [IE-4; (56)], 29 forced-choice items, with higher scores reflecting a more internal locus of control.

*Psychopathology* was assessed with the German version (57) of the Strengths and Difficulties Questionnaire [SDQ; (58)] in self-report, a screening instrument for emotional and behavioral problems in child and adolescents. The total difficulties score was used as the outcome, summing the four problem subscales (emotional symptoms, conduct problems, hyperactivity/inattention, and peer problems), with higher scores indicating greater levels of psychopathology.

with higher scores indicating greater levels of psychopathological symptoms.

### 2.4 Procedure

The present study is part of a prospective longitudinal project that initially recruited 101 mother-infant dyads between April 2002 and October 2003 at maternity wards in Germany.

Perinatal medical and psychological data were collected at baseline and all participants gave written informed consent for participation and recontact in later follow-ups. Follow-up assessments were carried out when the children were 2 weeks, 6 weeks, 4 months, 5.5 years, 14 years and 18–19 years old. During the first four waves (T1–T4), mothers reported on their own mental health and bonding experiences. At preschool age (T5), mothers provided information on children's social and temperamental characteristics. At T6, adolescents completed a structural clinical interview and questionnaires administered by a trained psychologist. At the final follow-up (T7), now young adults were invited to complete a set of online self-report measures targeting resilience, identity, and mental health.

For the T7 assessment, participants were contacted by email and received study information, an informed consent form, an individual study ID, and a personalized link to an online survey platform (SoSci Survey). The study was approved by the Ethics Committee of the Medical Association of Saarland (Ärztekammer des Saarlandes). After providing written consent, participants accessed the questionnaires using their ID number. All data were pseudonymized, and withdrawal from the study was possible at any time. On request, participants could alternatively complete the survey using paper questionnaires returned by mail.

### 2.5 Data analysis

All statistical analyses were conducted using R [Version 4.5.1; (59)]. Data manipulation and management were performed using dplyr [Version 1.1.4; (60)], tidyr [Version 1.3.1; (61)], reshape2 [Version 1.4.4; (62)]. Descriptive and inferential statistical analyses, including regression models, factor analyses, and correlation analyses, were conducted using car [Version 3.1-3; (63)], lm.beta [Version 1.7-2; (64)], lmtest [Version 0.9-40; (65)], psych [Version 2.5.6; (66)], GPArotation [Version 2025.1-3; (67)] and Hmisc [Version 5.2-3; (68)]. Data visualization was carried out using ggplot2 [Version 4.0.0; (69)], corrplot [Version 0.95; (70)] and RColorBrewer [Version 1.1-3; (71)].

Prior to statistical analyses, the dataset was examined for missing values and univariate outliers. For variables with partial missing values, different procedures were applied depending on the construct. For multi-item scales, “missing items” refers to individual items within a scale that were not answered by a participant. For multi-item scales with a small number of missing items scale scores were computed if at least 80% of items were available, following standard psychometric guidelines for handling missing items (72). For variables measured across multiple time points, mean scores were calculated across available timepoints, ignoring missing values, to preserve as much date as possible while maintaining reliability of the aggregated score.

Relevant variables with missing values were bonding subscales (T1 = 17.6%, T2 = 3.9%), maternal psychopathology (T5 = 2.0%) and social competences (T5 = 7.8%). For variables measured across time points, maternal mental health was averaged over the first five measure points (T1–T5), and bonding variables were averaged over the first four measurement points (T1–T4). Participants with missing data in the social competence scales (T5) were excluded from analyses including these variables.

Univariate outliers were identified using the ± 3 standard deviation (*SD*) criterion. Values exceeding three *SD*s above or below the mean were considered outliers. To reduce the influence of extreme values, these outliers were winsorized. In the present dataset, only a small number of values were affected by adjustment (bonding fear and care = 1 value, bonding risk and maltreatment = 2 values, maternal psychopathology T1–T4 mean = 1 value). Descriptive statistics and correlations are reported from the original unadjusted data. Regression models were computed using the cleaned dataset in which outliers were winsorized and missing values handled as above.

For H1, psychopathology at T7 was treated as the outcome predicted by the current psychological resources, whereas for H2, individual psychological resources at T7 were treated as outcomes predicted by early child, maternal and social factors. This distinction reflects the difference between protective effects on current mental health (H1) and developmental antecedents of resources (H2).

To address the issue of multiple testing within hypothesis block H1 and H2, a Benjamini-Hochberg false discovery rate [FDR; (73)] correction was applied to the six primary predictors (resilience, sense of coherence, optimism, internal locus of control, external locus of control, and self-efficacy). The correction was applied only to main predictors of theoretical interest and not to covariates or model comparisons tests. Results are reported with both unadjusted and FDR-adjusted *p*-values.

### 2.6 Risk of bias

To minimize bias, the study was pre-registered on AsPredicted before analysis, which reduces selective reporting. Standardized, validated instruments were applied consistently across assessment waves, data collection followed uniform procedures, to limit measurement and procedural bias. Missing data were handled transparently using mean aggregation across available timepoints or were excluded from analyses where necessary, reducing bias from incomplete data. To address potential inflation of Type I error due to multiple testing, false discovery rate (FDR) correction was applied. However, given the modest sample size, statistical power was limited, and effect estimates may be less precise. Findings, particularly for secondary outcomes and un-preregistered analyses, should therefore be interpreted cautiously.

## 3 Results

### 3.1 Descriptive statistics

The sample was approximately balanced by gender with 25 (49%) male and 26 (41%) female participants. Maternal education was high, with most mothers at T1 reporting a university or college degree (56.9%), followed by high school (25.5%), and secondary school (17.6%). Means, standard deviations, and spearman rank-order correlations for all study variables can be found in Supplementary Table S1 (Supplementary Material).

Psychological resources (resilience, sense of coherence, self-efficacy, and optimism) were positively correlated, while psychopathology at T6 and T7 showed negative associations with these resources. Peer-related social competence correlated negatively with adolescent psychopathology at T7, and higher maternal education was linked to lower psychopathology. Higher perceived risk of bonding maltreatment and impairment were negatively related to self-efficacy and optimism.

### 3.2 H1: associations with mental health at T7

To test the association between psychological resources and mental health at T7 all continuous predictor variables were standardized using *z*-transformation to allow direct comparison of regression coefficients across variables and to improve model interpretability. Maternal education was mean-centered to reduce multicollinearity with the intercepts, and adolescent sex was contrast coded (–1 = male, 1 = female) to allow interpretation of coefficients as the mean difference between males and females.

Regression analyses were conducted in a two-step procedure. First, unadjusted models were computed with each psychological resource measured at T7 (resilience, sense of coherence, optimism, locus of control, and self-efficacy) as predictors and psychopathological symptoms at T7 as outcome. Second, adjusted models were computed, additionally including adolescent sex (T1), maternal education (T1), prior adolescent psychopathology (T6) and maternal psychopathology (mean across T1–T5) as covariates to examine cross-sectional associations.

In the last step, the unadjusted and controlled models were compared using likelihood ratio tests (ANOVA) to assess whether including covariates significantly improved model fit. In all reported models, assumptions of linearity, homoscedasticity, normality of residuals, and multicollinearity were checked (e.g., QQ-plots, Shapiro–Wilk tests, and Breusch-Pagan tests). Model fit improvement compared to the null model was tested using ANOVA. The best models can be found in Table 2.

**Table 2:** H1: linear regression results predicting adolescent psychopathology at T7.

Predictor	Estimate	Std. Coeff.	95% CI	*t*-value	*p*-value
H1a Model with resilience					
Resilience	−1.60	−0.30	[–2.84, –0.37]	−2.61	.012*
Adolescent sex	0.53	0.10	[–0.53, 1.58]	1.00	.321
Maternal education T1	−1.72	–0.25	[–3.08, –0.36]	−2.55	.014*
Adolescent psychopathology T6	2.30	0.44	[1.11, 3.49]	3.89	<.001***
Maternal psychopathology T1–T5	0.48	0.09	[–0.62, 1.57]	0.88	.385
H1b Model with sense of coherence (SOC)					
SOC	–3.29	–0.62	[–4.35, –2.23]	–6.23	<.001***
Adolescent sex	0.84	0.16	[−0.00, –2.23]	2.01	.050
Maternal education T1	–1.13	–0.17	[−2.22, –0.04]	–2.08	.043*
Adolescent psychopathology T6	1.13	0.21	[0.10, 2.16]	2.21	.032*
Maternal psychopathology T1–T5	0.46	0.09	[–0.36, 1.29]	1.11	.274
H1c Model with optimism					
Optimism	–1.60	–0.30	[–2.76, –0.45]	–2.81	.007**
Adolescent sex	0.62	0.12	[–0.44, 1.67]	1.18	.242
Maternal education T1	–1.61	–0.24	[–2.97, –0.26]	–2.40	.021*
Adolescent psychopathology T6	2.35	0.45	[1.20, 3.50]	4.12	<.001***
Maternal psychopathology T1–T5	0.95	0.18	[–0.09, 1.98]	1.85	.072
H1d Model with locus of control					
Internal locus of control	–0.62	–0.12	[–1.84, 0.59]	–1.04	.304
External locus of control	1.21	0.23	[–0.05, 2.46]	1.94	.059
Adolescent sex	0.36	0.07	[–0.69, 1.42]	0.70	.490
Maternal education T1	–1.18	–0.17	[–2.65, 0.30]	–1.61	.115
Adolescent psychopathology T6	2.75	0.52	[1.64, 3.86]	4.98	<.001***
Maternal psychopathology T1–T5	0.48	0.09	[–0.62, 1.59]	0.88	.383
H1e Model with self-efficacy					
Self-efficacy	−1.29	–0.25	[–2.41, –0.17]	–2.32	.025*
Adolescent sex	0.30	0.06	[–0.75, 1.36]	0.58	.567
Maternal education T1	−1.53	–0.23	[–2.94, –0.13]	–2.20	.033*
Adolescent psychopathology T6	2.71	0.51	[1.60, 3.81]	4.92	<.001***
Maternal psychopathology T1–T5	0.79	0.15	[–0.27, 1.86]	1.50	.140

*N* = 51. Std. Coeff., standardized coefficients; CI, confidence interval; Continuous predictors were standardized (*z*-transformation), maternal education was mean-centered, and adolescent sex was contrast coded (–1 = male, 1 = female).

* *p* < .05.

** *p* < .01.

*** *p* < .001.

#### 3.2.1 H1a resilience

Higher resilience predicted lower psychopathological symptoms, b = –2.92, *SE* = 0.63, *t*(49) = –4.66, *p* < .001 (*R*^2^ = .31, adjusted *R*^2^ = .29). When controlling for sex, maternal education, prior adolescent psychopathology, and maternal psychopathology, resilience remained a significant predictor. Maternal education was associated with fewer symptoms and prior psychopathology at T6 predicted higher symptom levels (*R*^2^ = .57, adjusted *R*^2^ = .52). An ANOVA comparing the unadjusted and controlled model confirmed that adding covariates significantly improved model fit, *F*(4, 45) = 6.84, *p* < .001.

#### 3.2.2 H1b sense of coherence (SOC)

A stronger SOC predicted lower psychopathological symptoms, b = –4.19, *SE* = 0.46, *t*(49) = –9.16, *p* < .001 (*R²* = .63, adjusted *R*^2^ = .62). The effect remained robust after adding covariates, with maternal education negatively and prior adolescent psychopathology positively predicting symptom levels (*R*^2^ = .73, adjusted *R*^2^ = .70). An ANOVA comparing the unadjusted and controlled models indicated that including the covariates significantly improved model fit, *F*(4, 45) = 4.33, *p* = .005.

#### 3.2.3 H1c optimism

Higher optimism was linked to fewer psychopathological symptoms, b = –2.65, *SE* = 0.65, *t*(49) = –4.07, *p* < .001 (*R*^2^ = .25, adjusted *R*^2^ = .24). The effect remained significant in the controlled model, along with maternal education as a negative and prior adolescent psychopathology as a positive predictor (*R*^2^ = .58, adjusted *R*^2^ = .53). An ANOVA comparing the unadjusted and controlled model confirmed that adding the covariates significantly improved model fit, *F*(4, 45) = 8.65, *p* < .001.

#### 3.2.4 H1d locus of control

An internal locus of control predicted lower psychopathology, b = –1.60, *SE* = 0.72, *t*(48) = –2.22, *p* < .031, while external control predicted higher psychopathology, b = 1.60, *SE* = 0.72, *t*(48) = 2.22, *p* < .031 (*R*^2^ = .26, adjusted *R*^2^ = .23). After covariate adjustment, both effects became nonsignificant, whereas prior adolescent psychopathology remained the only significant predictor (*R*^2^ = .57, adjusted *R*^2^ = .51). An ANOVA comparing unadjusted and controlled models indicated a significant improvement in model fit for the controlled model, *F*(4, 44) = 7.93, *p* < . 001.

#### 3.2.5 H1e self-efficacy

Higher self-efficacy predicted lower psychopathology, b = –2.36, *SE* = 0.67, *t*(49) = –3.51, *p* < .001 (*R*^2^ = .20, adjusted *R*^2^ = .18). The association remained significant in the controlled model, together with maternal education as a negative and adolescent psychopathology as a positive predictor (*R*^2^ = .56, adjusted *R*^2^ = .51). An ANOVA comparing the unadjusted and controlled model indicated that adding covariates significantly improved model fit, *F*(4, 45) = 9.04, *p* < .001.

After applying Benjamini-Hochberg FDR correction across the six H1 predictors, associations for resilience (*p _FDR_* = .024), sense of coherence (*p _FDR_* =  < .001), optimism (*p _FDR_* = .022), and self-efficacy (*p _FDR_* = .037) remained significant. Internal and external locus of control did not survive correction (*p _FDR_* > .05).

#### 3.2.6 Exploratory and un-preregistered multiple regression

When all five psychological resources were entered simultaneously, the model was significant, *F*(6, 44) = 13.76, *p* < .001 (*R*^2^ = .65, adjusted *R*^2^ = .60). Only sense of coherence emerged as a significant negative predictor of psychopathological symptoms (b = –4.42, *SE* = 0.90, *t* = –4.92, *p* < .001), while none of the other factors were significant. The controlled model including all covariates (see Table 3) remained significant, *F*(10, 40) = 11.82, *p* < .001 (*R*^2^ = .74, adjusted *R*^2^ = .68), with SOC as significant negative predictor and prior adolescent psychopathology at T6 as a significant positive predictor. An ANOVA confirmed that including the covariates significantly improved model fit, *F*(10, 40) = 11.82, *p* < .001.

**Table 3:** Linear regression results predicting adolescent psychopathology at T7.

Predictor	Estimate	Std. Coeff.	95% CI	*t*-value	*p*-value
Resilience	0.62	0.12	[–1.17, 2.41]	0.70	.488
Sense of coherence	–3.57	–0.68	[–5.31, –1.82]	–4.13	<.001***
Optimism	0.15	0.03	[–1.04, 1.34]	0.26	.798
External Locus of Control	0.29	0.05	[–0.87, 1.47]	0.50	.622
Internal Locus of Control	0.50	0.09	[–0.62, 1.62]	0.90	.376
Self-Efficacy	–0.37	–0.07	[–1.93, 1.19]	–0.48	.635
Adolescent sex	0.78	0.15	[–0.11, 1.67]	1.77	.085
Prior child psychopathology (T6)	1.26	0.24	[0.17, 2.35]	2.34	.024*
Mat. education (T1)	–0.94	–0.14	[–2.14, 0.25]	–1.60	.118
Mat. psychopathology (T1–T5)	0.44	0.08	[–0.56, 1.43]	0.88	.383

*N* = 51. Std. Coeff., standardized coefficients; CI, confidence interval. Continuous predictors were standardized (*z*-transformation), maternal education was mean-centered, and adolescent sex was contrast coded (–1 = male, 1 = female).

* *p* < .05.

*** *p* < .001.

### 3.3 Exploratory factor analysis of psychological resources (un-preregistered)

To examine whether the five psychological resources represent distinct constructs or overlapping dimensions, an exploratory factor analysis (EFA) was conducted. Given the modest sample size and the exploratory aim of identifying the latent structure of closely related constructs, an EFA was considered more appropriate than a confirmatory factor analysis, which would have required substantially larger samples for stable parameter estimation.

Sampling adequacy was acceptable (KMO = 0.71, Bartlett's *x*^2^(15) = 298.13, *p* < .001), indicating that the data were suitable for factor analysis. Parallel analysis supported a single-factor solution, suggesting a strong general psychological resource factor capturing substantial shared variance across all five constructs.

**Figure 1:**
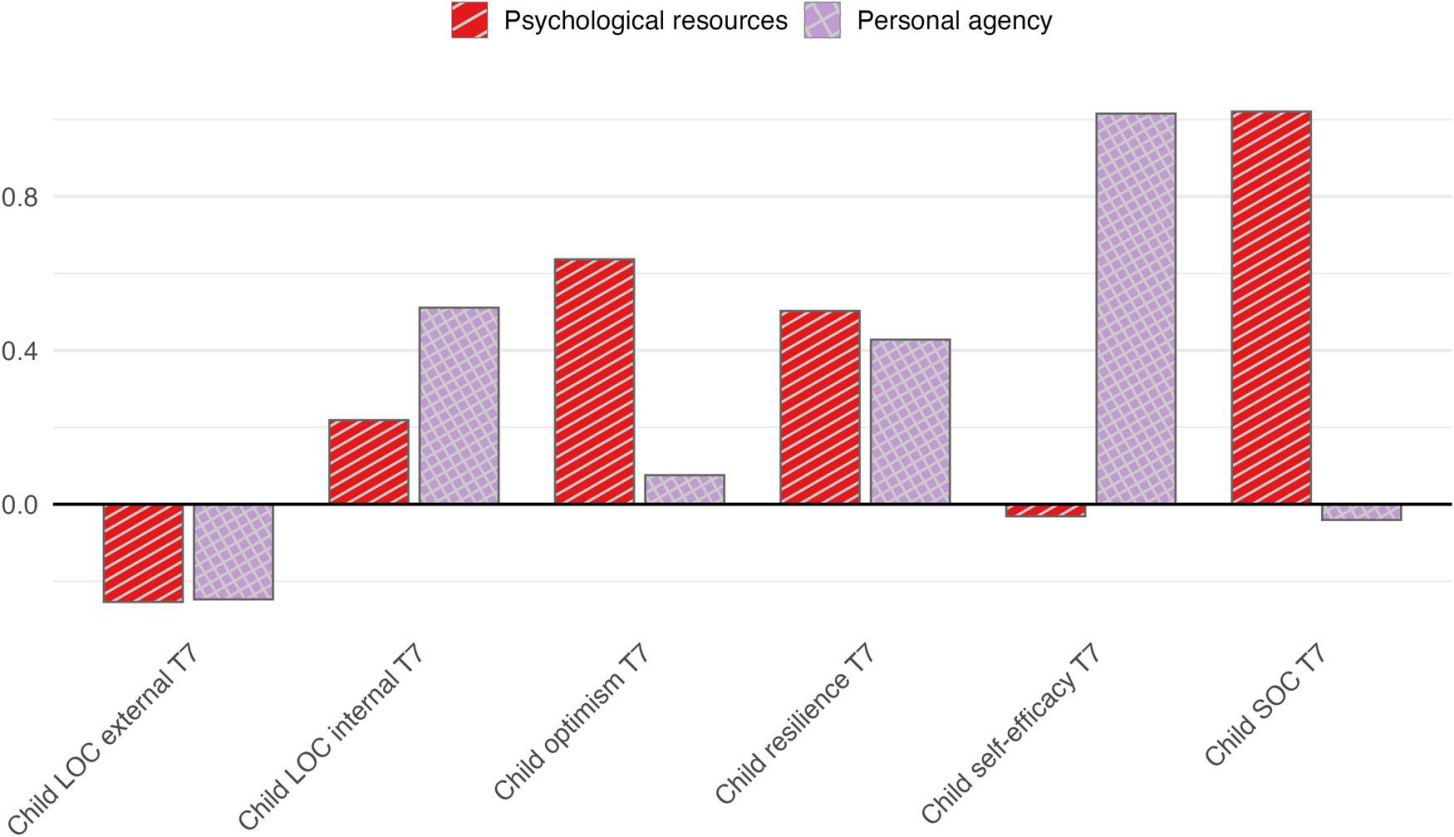
Exploratory factor analysis: factor loadings of the two-factor solution. Psychological resources = red and striped, Personal agency = purple and checked. The bar plot illustrates standardized factor loadings from the exploratory factor analysis of psychological resource variables assessed at T7. The secondary two-factor solution was extracted using maximum likelihood estimation with oblimin rotation and is presented for descriptive purposes.

To further explore potential subdimensions within this general factor, a secondary two-factor solution (maximum likelihood extraction, oblimin rotation) was additionally examined (Figure 1 and Table 4). This model yielded a general psychological resource factor, with substantial loadings for resilience, SOC, and optimism and a personal agency factor, with self-efficacy and internal locus of control loading predominantly here. External locus of control showed low and mixed loadings across both factors. The two factors were moderately correlated (*r* = 0.59). Model fit indices for the two-factor solution indicated moderate fit (RMSR = 0.06, TLI = 0.89, RMSEA = 0.13). According to conventional thresholds, RMSR ≤ 0.08 and TLI ≥ 0.90 suggest acceptable fit, while RMSEA ≤ 0.06 indicates good fit (74). The observed elevated RMSEA should be interpreted cautiously given the small sample size and low degrees of freedom (75). Overall, the results indicate a dominant general psychological resource factor with partial differentiation into agency-related components. Given the strong general factor, moderate factor correlation, and limited sample size, factor score regressions were not conducted.

**Table 4:** Exploratory and descriptive results of factor analysis of psychological resources.

Variable	Factor 1	Factor 2	*h^2^*	*u^2^*	*com*
Resilience	0.50	0.43	0.69	0.31	1.95
Sense of Coherence	1.02	−0.04	1.00	0.00	1.00
Optimism	0.64	0.08	0.47	0.53	1.03
Self-Efficacy	−0.03	1.02	1.00	0.00	1.00
Internal Locus of Control	0.22	0.51	0.44	0.56	1.35
External Locus of Control	−0.25	−0.25	0.20	0.80	2.00

Factor 1 represents a common psychological resources factor. Factor 2 represents a partially independent personal agency factor. Loadings are standardized pattern matrix values from EFA (maximum likelihood extraction, oblimin rotation). Communality (*h*^2^), uniqueness (*u*^2^), and factor complexity (*com*) are reported. The two factors were moderately correlated (*r* = 0.59). Given the small sample size, standard errors and *p*-values for loadings are not reported.

### 3.4 H2. Early predictors of psychosocial resources (exploratory and pre-registered)

Linear regressions were computed for each psychological resource [standardized predictors; covariates included in the base model: adolescent sex T1, adolescent psychopathology (T6) maternal education T1, maternal psychopathology T1–T5]. In additional exploratory models, early child social competences (self-oriented, other, oriented, and peer relations at T5) and bonding dimensions (bonding impairment, rejection/anger, fear of care, and risk of maltreatment, means across T1–T4) were entered to examine further variance explained. Model assumptions (linearity, homoscedasticity, and normality of residuals, multicollinearity) were checked using QQ-plots, Shapiro–Wilk tests, and Breusch-Pagan tests. Improvements in model fit were evaluated using analyses of variance (ANOVA). The best models are reported in Table 5.

**Table 5:** H2: linear regression results predicting psychological resources at T2.

Predictor	Estimate	Std. Coeff.	95% CI	*t*-value	*p*-value
H2a Resilience					
Adolescent psychopathology T6	−0.43	−0.43	[–0.68, –0.17]	–3.36	.002**
Adolescent sex	0.14	0.14	[–0.11, 0.39]	1.11	.272
Mat. education T1	0.11	0.08	[–0.21, 0.44]	0.69	.496
Mat. Psychopathology (T1–T5)	–0.26	–0.26	[–0.51, –0.01]	–2.11	.040*
H2b Sense of coherence					
Adolescent psychopathology T6	–0.56	–0.56	[–0.80, –0.33]	–4.84	<.001***
Adolescent sex	0.16	0.16	[–0.07, 0.39]	1.42	.162
Mat. education T1	0.23	0.18	[–0.07, 0.53]	1.58	.120
Mat. Psychopathology (T1–T5)	–0.13	–0.13	[–0.36, 0.10]	–1.18	.246
H2c Optimism					
Adolescent psychopathology T6	–0.39	–0.39	[–0.66, –0.12]	–2.91	.005**
Adolescent sex	0.20	0.20	[–0.07, 0.46]	1.50	.141
Mat. education T1	0.17	0.14	[–0.17, 0.52]	1.02	.314
Mat. Psychopathology (T1–T5)	0.03	0.03	[–0.24, 0.30]	0.24	.818
H2d Internal locus of control^a^					
Adolescent psychopathology T6	–0.25	–0.25	[–0.52, 0.03]	–1.78	.081
Adolescent sex	0.02	0.02	[–0.25, 0.29]	0.15	.884
Mat. education T1	0.34	0.26	[–0.01, 0.69]	1.93	.059
Mat. Psychopathology (T1–T5)	–0.10	–0.10	[–0.37, 0.17]	–0.76	.454
H2d External locus of control					
Adolescent psychopathology T6	0.06	0.06	[–0.21, 0.33]	0.47	.643
Adolescent sex	–0.04	–0.04	[–0.30, 0.22]	–0.30	.765
Mat. education T1	–0.42	–0.33	[–0.76, –0.08]	–2.47	.017*
Mat. Psychopathology (T1–T5)	0.29	0.29	[0.03, 0.55]	2.21	.032*
H2e Self-efficacy					
Adolescent psychopathology T6	–0.24	–0.24	[–0.51, 0.04]	–1.74	.089
Adolescent sex	0.01	0.00	[–0.26, 0.27]	0.04	.972
Mat. education T1	0.44	0.34	[0.07, 0.81]	2.42	.020*
Mat. Psychopathology (T1–T5)	0.13	0.13	[–0.17, 0.42]	0.88	.386
Bonding impairment (T1–T4)	–0.09	–0.09	[–0.50, 0.33]	–0.43	.670
Bonding rejection anger (T1–T4)	–0.11	–0.11	[–0.46, 0.25]	–0.60	.552
Bonding fear care (T1–T4)	0.05	0.05	[–0.29, 0.39]	0.30	.770
Bonding risk maltreat. (T1–T4)	–0.42	–0.42	[–0.75, –0.08]	–2.52	.016*

*N* = 51. Std. Coeff., standardized coefficients; CI, confidence interval. Continuous predictors were standardized (*z*-transformation), maternal education was mean-centered, and adolescent sex was contrast coded (–1 = male, 1 = female).

^a^ The base model did not differ significantly from the null model but is reported for transparency and comparability across models.

* *p* < .05.

** *p* < .01.

*** *p* < .001.

Because of missing values in the social competence variables, four cases were removed from the extended analyses. Therefore, the final model was reported using the full sample, whereas model comparisons were based on the reduced sample.

#### 3.4.1 H2a resilience

The base model was significant, *F*(4, 46) = 4.75, *p* = .003, (*R*^2^ = .29, adjusted *R*^2^ = .23), while adding early social competences, Δ*F*(3, 39) = 0.51, *p* = .680, and bonding dimensions, Δ*F*(4, 38) = 1.62, *p* = .190, did not result in a significant model improvement. In the final model, higher adolescent psychopathology at T6 predicted lower resilience at T7 (b = –0.43, *p* = .002). In addition, higher maternal psychopathology across T1–T5 significantly predicted lower resilience (b = –0.26, *p* = .040). Adolescent sex and maternal education were not statistically significant predictors.

#### 3.4.2 H2b sense of coherence (SOC)

The base model was significant, *F*(5, 46) = 7.94, *p* < .001 (*R*^2^ = .41, adjusted *R*^2^ = .36). Adding early social competences, Δ*F*(3, 39) = 0.93, *p* = .435, and bonding dimensions, Δ*F*(4, 38) = 0.91, *p* = .466, did not improve model fit. In the final model, higher adolescent psychopathology at T6 predicted lower SOC at T7 (b = –0.56, *p* < .001), adolescent sex, maternal education, and maternal psychopathology across T1–T5 were not statistically significant predictors.

#### 3.4.3 H2c optimism

The base model was significant, *F*(4, 46) = 2.90, *p* < .032 (*R*^2^ = .20, adjusted *R*^2^ = .13). Adding early social competences, Δ*F*(3, 39) = 1.86, *p* = .152, and bonding dimensions, Δ*F*(4, 38) = 1.64, *p* = .184, did not improve model fit. In the final model, higher adolescent psychopathology at T6 predicted lower optimism at T7 (b = –0.39, *p* = .005), while other predictors were not significant.

#### 3.4.4 H2d locus of control

For internal locus of control at T7, the base model approached significance, *F*(4, 46) = 2.26, *p* = .078 (*R*^2^ = .16, adjusted *R*^2^ = .09). Adding social competences, Δ*F*(3, 39) = 0.75, *p* = .528, or bonding dimensions, Δ*F*(4, 38) = 1.48, *p* = .228, did not significantly improve model fit. Further, none of the predictors reached statistical significance in the final model.

For external locus of control at T7, the base model was statistically significant, *F*(4, 46) = 3.10, *p* = .024 (*R*^2^ = .21, adjusted *R*^2^ = .14). Adding bonding dimensions did not result in a significant model improvement, Δ*F*(3, 39) = 1.52, *p* = .224, and adding bonding dimensions also did not improve the model, Δ*F*(3, 38) = 1.14, *p* = .353. In the final model, maternal education (T1) was negatively associated with external locus of control (b = –0.42, *p* = .017), and maternal psychopathology (T1–T5) was positively associated (b = –0.29, *p* = .032). Other predictors did not reach significance.

#### 3.4.5 H2e self-efficacy

For self-efficacy at T7 the base model was not statistically significant, *F*(4, 42) = 1.77, *p* = .152 (*R*^2^ = .14, adjusted *R*^2^ = .06). Adding social competence variables to the model did not significantly improve model fit, Δ*F*(3, 39) = 0.46, *p* = .713, but adding bonding dimensions improved model fit, Δ*F*(3, 38) = 3.15, *p* = .025. In the final model with the bonding dimensions, *F*(8, 42) = 2.32, *p* = .037 (*R*^2^ = .31, adjusted *R*^2^ = .17), maternal education (b = 0.44, *p* = .020) was a significant positive predictor and bonding risk/maltreatment (b = –0.42, *p* = .016) a significant negative predictor, while other predictors were not significant.

After applying Benjamini-Hochberg FDR correction across the six developmental models, adolescent psychopathology at T6 remained a significant predictor of resilience (*p _FDR_* = .005), sense of coherence (*p _FDR_* =  < .001), and optimism (*p _FDR_* = .011). Other associations with internal locus of control, external locus of control, and self-efficacy where either not significant in the uncorrected analysis or did not remain significant after correction (*p _FDR_* > .05). The effect of bonding risk/maltreatment (*p _FDR_* = .039) remains robust after FDR correction.

## 4 Discussion

### 4.1 Summary results

The present study investigated the associations between psychological resources in young adulthood and psychopathology, as well as early predictors of these resources.

#### 4.1.1 H1: associations with mental health

As hypothesized, higher levels of resilience, sense of coherence, optimism, and self-efficacy were associated with lower psychopathology symptoms in young adulthood, even after controlling for adolescent sex, maternal education, prior adolescent psychopathology, and maternal psychopathology. Locus of control showed expected effects in unadjusted models, but these associations were no longer significant after accounting for covariates. Exploratory regression including all psychological resources simultaneously showed that sense of coherence uniquely predicted current psychopathology, highlighting its central role in mental health outcomes. Multiple testing was addressed using a Benjamini–Hochberg false discovery rate (FDR) correction across the six primary predictors. Findings remained largely robust after FDR adjustment.

An explanatory factor analysis indicated substantial overlap among the assessed psychological resources, supporting a predominant single-factor solution reflecting a general psychological resource dimension. This factor captured the majority of shared variance across resilience, sense of coherence, optimism, self-efficacy, and locus of control measures. For descriptive purposes, an alternative two-factor solution was additionally examined to explore potential differentiation within this general resource construct. In this solution, resilience, sense of coherence, optimism, loaded primarily on the general psychological resource factor, whereas self-efficacy and internal locus of control formed a related but partially distinct personal agency factor. External locus of control showed low and inconsistent loadings across both factors. Given the exploratory nature of the factor analysis, the modest sample size, and the limited statistical power to formally compare competing models, the one-factor solution should be regarded as the primary structural representation, whereas the two-factor solution is best interpreted as exploratory.

#### 4.1.2 H2: early predictors of psychological resources

Across psychological resources at T7, adolescent psychopathology at T6 emerged as the most consistent predictor, with higher levels reporting lower resilience, sense of coherence, optimism, self-efficacy, and a more external locus of control in young adulthood. After FDR correction, adolescent psychopathology at T6 remained significant for resilience, SOC, and optimism only. Maternal education (T1) and maternal psychopathology (T1–T5) showed selective and inconsistent associations, contributing primarily to resilience and external locus of control. Early social competences did not add explanatory value beyond the base models. Notably, bonding risk/maltreatment significantly predicted self-efficacy, such that higher perceived risk was associated with lower self-efficacy, and this effect remained significant after FDR correction. In contrast, other bonding and social competence variables did not explain additional variance.

Overall, the findings suggest that psychological resources in young adulthood are closely linked to both prior and current mental health, with SOC emerging as a particularly salient resource within this pattern of associations.

### 4.2 Interpretation and theoretical implications

The present findings contribute to a growing body of evidence on the developmental interplay between psychological resources and mental health by demonstrating associations between multiple resource constructs and psychopathology in young adulthood (18–19 years). Higher levels of resilience, sense of coherence (SOC), optimism, and self-efficacy were linked with lower levels of psychopathological symptoms, extending previous evidence on their relevance for mental health (14–17).

Among the assessed resources, SOC showed the strongest association with psychopathology, consistent of prior work identifying SOC as a central correlate of mental health (76) and with Antonovsky's salutogenetic framework, in which SOC is conceptualized as an overarching orientation that shapes how individuals perceive, manage, and find meaning in stressors (13). Rather than representing a single coping skill, SOC may reflect an integrative framework through which other psychological resources may be organized and mobilized.

While these patterns are consistent with transactional models proposing reciprocal and context-sensitive relations between psychological resources and mental health over time (77–79), the concurrent assessment of resources and psychopathology in young adulthood precludes conclusions about directionality.

The exploratory factor analysis supported this interpretation by indicating substantial shared variance across psychological resources, with a dominant general factor likely capturing broad adaptive orientations such as perceived coherence, manageability, and confidence in coping with environmental demands. A secondary two-factor solution suggested partial differentiation between general resources and a personal-agency component, but this structure was examined for descriptive purposes only and needs to be investigated in further studies.

Regarding developmental influences, psychological resources in young adulthood are closely linked to prior mental health. Adolescent psychopathology at 14 years was consistently associated with lower resilience, sense of coherence, and optimism at 18–19 years, even after correction for multiple testing, converging with prior evidence linking ongoing symptomatology to diminished psychological resources (17, 76, 80, 81). Associations with internal and external locus of control and self-efficacy did not survive correction, indicating that their observed effects may reflect chance or smaller, less reliable effects.

Beyond mental health, higher early bonding risk indicative of maltreatment within the first 14 months of life was associated with lower self-efficacy in young adulthood, suggesting that early experiences of insecure bonding may undermine beliefs in personal agency and environmental reliability (82). In contrast, other bonding factors or social competences at 5.5 years showed no significant explanatory value, underscoring the specificity rather than generality of early interpersonal effects.

Contrary to prior findings (83–85), maternal education at childbirth and maternal psychopathology during the first 5.5 years had only limited and inconsistent effects, suggesting that later adolescent outcomes may be increasingly shaped by the adolescent's own mental health rather than by early caregiving factors alone. This likely reflects the low levels of maternal psychopathology in this non-clinical sample (mean T1–T4 = 0.23 on the SCL-90-R), resulting in a restricted range. Adolescent psychopathology at T6 (mean T6 = 9.25 on the BSI-18) and T7 (mean T7 = 10.14 on the SDQ) was also low, further limiting variability. Maternal factors may still operate indirectly, moderating the relationship between adolescent psychopathology and later resource development, consistent with their role in early affect regulation and attachment security (86, 87). Over time, the impact of these early maternal influences on later psychological resources might appear to be increasingly mediated by the adolescent's own regulatory capacities and social experiences, highlighting the dynamic, context-dependent nature of resource development (88).

Importantly, this study addresses a gap by examining how early influences relate to the co-development of multiple psychological resources and their links with psychopathology in young adulthood. By analysing these resources simultaneously, our findings suggest that they are closely interconnected, with SOC playing a particularly central integrative role in this sample.

### 4.3 Practical and clinical implications

Although the present findings are based on observational data and do not allow causal conclusions, they offer several implications for preventive and clinical practice. The consistent associations between multiple psychological resources—particularly sense of coherence (SOC), resilience, and optimism—and lower levels of psychopathology suggest that resource-oriented perspectives may be useful frameworks for understanding mental health in adolescence and young adulthood.

Rather than focusing on isolated traits, the predominance of a general psychological resource dimension points to the potential relevance of interventions that address interconnected adaptive orientations, such as comprehensibility, manageability, and meaningfulness, which represent the core components of sense of coherence. Resource-oriented approaches may therefore benefit from targeting integrated belief systems and coping frameworks through strategies such as metacognitive training, narrative reconstruction, structured reflection, role-play, or emotion regulation training (89–93).

The strong cross-sectional association between current psychopathology and diminished psychological resources highlights the importance of considering psychological resources within ongoing clinical care. While psychological resources may support adaptive coping, elevated psychopathology may concurrently limit access to or utilization of these resources. Interventions should adopt a bidirectional approach, combining symptom-focused therapy (e.g., CBT for anxiety or depression) with coping-skills training that strengthens adaptive beliefs and meaning-making (94).

Findings related to specific resource domains further suggest differentiated implications. The association between early maltreatment risk and lower self-efficacy indicates that interventions addressing beliefs about personal agency may be especially relevant for individuals with adverse early experiences. However, given the limited and selective effects of caregiving and maternal factors in the present study, conclusions regarding targeted early prevention should be drawn cautiously.

Moreover, the present findings do not demonstrate that psychological resources are modifiable or that their enhancement leads to long-term improvements in mental health. Rather, they support the assumption that psychological resources remain closely linked to current mental health in young adulthood. The limited predictive role of early maternal factors hints that psychological resources are experience-sensitive and context-dependent rather than fixed by early environmental influences.

From a practical perspective, integrating resource-oriented approaches into educational, clinical, and community settings may therefore support both preventive and rehabilitative functions, particularly combined with symptom-focused interventions. Longitudinal-intervention studies are needed to determine whether strengthening key psychological resources can causally influence later psychopathology trajectories.

### 4.4 Limitations

Several limitations should be noted when interpreting these findings.

First, although the longitudinal design provides valuable insight into developmental processes, causality cannot be drawn due to potential bidirectional influences between current adolescent psychopathology and psychological resources, both measured at the same timepoint (95).

Second, most measures relied on self-report, increasing susceptibility to recall bias and social desirability effects (96). In addition, measurement instruments differed across time points. Adolescent psychopathology at T6 was parent-reported, whereas T7 assessments were self-reported, potentially contributing to discrepancies that reflect informant-related variance rather than true developmental change. Parent-adolescent agreement was moderate, with an intraclass correlation coefficient of ICC = 0.62 [95% CI (0.42, 0.77), *p* < .001], indicating that some differences between T6 and T7 scores may reflect differences in informant perspective.

Third, although the factor analysis supported a meaningful clustering of psychological resources, shared method variance and conceptual overlap could inflate intercorrelations. Multicollinearity was controlled in all regression models, suggesting that the observed effects represent partially distinct components of a broader resource system rather than methodological artifacts. To address the issue of multiple testing, Benjamini-Hochberg FDR corrections were applied separately for the H1 and H2 hypothesis blocks. After correction, only resilience, sense of coherence, and optimism remained significant developmental predictors, whereas associations with internal and external locus of control and self-efficacy did not survive, highlighting that some observed effects may reflect chance or smaller, less reliable associations. The relatively modest sample size limits statistical power, allowing only medium-to-large effects to be reliably detected, which should be considered when interpreting null findings, especially for bonding and maternal psychopathology. Furthermore, some constructs, such as bonding and maternal psychopathology, were represented by mean scores across time points to reduce model complexity. Internal consistency was high for maternal psychopathology (Cronbach's α = .78–.86), but only moderate for bonding measures (Cronbach's α = .43–.88), limiting the interpretability of null findings in this domain.

Fourth, the long intervals between measurement waves may obscure micro-level developmental processes. Future studies could benefit from shorter, more fine-grained longitudinal designs with dynamic modeling (e.g., cross-lagged panel) to capture reciprocal influences between resources and psychopathology.

Fifth, sample characteristics may limit generalizability. The cohort was relatively homogeneous, well-educated, and of high socioeconomic status (97). Although sample size (*N* = 51) was adequate for the regression analyses and exploratory factor analysis conducted, but it may have limited power to detect small effects or subtle interactions. Attrition over time could have introduced bias, as we did not examine whether participants retained at T7 differed systematically from those lost to follow-up on early risk variables, because the relevant data for such comparisons were not available. Replication in diverse populations, clinical samples, and different cultural contexts is warranted.

Finally, future research should adopt process-oriented, multi-informant approaches to improve construct validity and disentangle shared variance across assessments (98). Incorporating behavioral, physiological, or implicit measures of psychological resources, as well as longitudinal-intervention designs, could help test whether targeted enhancement of key psychological resources alters subsequent psychopathology trajectories. Examining reciprocal dynamics across sensitive developmental transitions (e.g., late adolescence to adulthood) will further clarify the mechanisms underlying resource development and mental health outcomes.

## 5 Conclusion

This study adds further evidence for the close association between psychological resources—particularly sense of coherence, resilience, optimism, and self-efficacy—and mental health in young adulthood. SOC emerged as a meta-resource, integrating adaptive beliefs about coherence, competence, and coping across life domains. The findings support a dynamic, multidimensional framework, in which psychological resources co-develop and are closely intertwined with mental health.

Exploratory factor analyses indicated substantial shared variance among these resources, with a dominant general factor and limited evidence for partial differentiation related to personal agency, suggesting that psychological resources constitute interconnected components of a broader adaptive system rather than fully independent constructs.

Psychological resources in young adulthood were linked to both prior and concurrent mental health. Adolescent psychopathology at 14 years emerged as the most consistent developmental predictor of later resilience, sense of coherence, and optimism, even after controlling for multiple testing. Associations with internal and external locus of control and self-efficacy were either non-significant or did not survive correction, indicating that some observed effects may reflect smaller or less reliable associations. In contrast, early maternal and caregiving factors showed only weak and selective associations, underscoring the context-dependent and non-linear nature of developmental influences on psychological resources across time.

However, these findings do not allow conclusions regarding causality, modifiability, or long-term effects. Rather, they highlight the central association between psychological resources and mental health during late adolescence and emerging adulthood. Understanding how these resources co-develop and interact could help identify potential targets for preventive interventions and the promotion of well-being. Future longitudinal intervention-based studies are needed to clarify whether and how strengthening key psychological resources can causally influence mental health trajectories across developmental transitions.

In sum, psychological resources appear to operate as an integrated, dynamic system in emerging adulthood, closely associated with mental health and selectively shaped by earlier developmental experiences. These findings underscore the theoretical and practical importance of considering psychological resources as interconnected components of well-being, offering opportunities to foster resilience, coping, and adaptive functioning in young people.

## Data Availability

The raw data supporting the conclusions of this article will be made available by the authors, without undue reservation.

## References

[1] Buhler-Wassmann AC Hibel LC. Studying caregiver-infant co-regulation in dynamic, diverse cultural contexts: a call to action. Infant Behav Dev. (2021) 64:101586. 10.1016/j.infbeh.2021.101586 34118652 PMC10314734

[2] Paley B Hajal NJ. Conceptualizing emotion regulation and coregulation as family-level phenomena. Clin Child Fam Psychol Rev. (2022) 25(1):19–43. 10.1007/s10567-022-00378-4 35098427 PMC8801237

[3] Brumariu LE. Parent–child attachment and emotion regulation. New Dir Child Adolesc Dev. (2015) 2015(148):31–45. 10.1002/cad.20098 26086126

[4] Shields A Cicchetti D. Emotion regulation among school-age children: the development and validation of a new criterion Q-sort scale. Dev Psychol. (1997) 33(6):906–16. 10.1037/0012-1649.33.6.906 9383613

[5] Zaki J Williams W. Interpersonal emotion regulation. Emotion. (2013) 13:803–10. 10.1037/a0033839 24098929

[6] Bowlby J. Attachment and Loss. New York: Basic Books (1969). (Attachment and Loss; Bd. 1). Available online at: https://books.google.de/books?id=FYEuAAAAMAAJ (Accessed September 09, 2026).

[7] Le Bas GA Youssef GJ Macdonald JA Rossen L Teague SJ Kothe EJ The role of antenatal and postnatal maternal bonding in infant development: a systematic review and meta-analysis. Soc Dev. (2020) 29(1):3–20. 10.1111/sode.12392

[8] Ranson K Urichuk L. The effect of parent-child attachment relationships on child biopsychosocial outcomes: a review. Early Child Dev Care. (2006) 178:129–52. 10.1080/03004430600685282

[9] Groh A van IJzendoorn M Bakermans-Kranenburg M Roisman G. Attachment in the early life course: meta-analytic evidence for its role in socioemotional development. Child Dev Perspect. (2016) 11:70–6. 10.1111/cdep.12213

[10] Hatakka E Flykt M Rusanen E Kylliäinen A Paavonen EJ Kiviruusu O. Longitudinal associations between parental bonding and child preschool social-emotional problems: the unique and combined role of mothers and fathers. Child Psychiatr Hum Dev. (2025). 10.1007/s10578-025-01886-4 40715671

[11] Rusanen E Lahikainen AR Vierikko E Pölkki P Paavonen EJ. A longitudinal study of maternal postnatal bonding and psychosocial factors that contribute to social-emotional development. Child Psychiatr Hum Dev. (2024) 55(1):274–86. 10.1007/s10578-022-01398-5 PMC10796530 35870058

[12] Joas J Moehler E. Maternal bonding in early infancy predicts childrens’ social competences in preschool age. Front Psychiatry. (2021) 12:687535. 10.3389/fpsyt.2021.687535 34489753 PMC8416914

[13] Antonovsky A. Unraveling the mystery of health: how people manage stress and stay well. J-B Soc Behav Sci Ser. (1987).

[14] Färber F Rosendahl J. The association between resilience and mental health in the somatically ill—a systematic review and meta-analysis. Dtsch Arztebl Int. (2018) 115:621–7. 10.3238/arztebl.2018.0621 30373706 PMC6218704

[15] Li X Xia B Shen G Dong R Xu S Yang L. The interplay of depressive symptoms and self-efficacy in adolescents: a network analysis approach. Front Psychol. (2024) 15:1419920. 10.3389/fpsyg.2024.1419920 39282676 PMC11393584

[16] Rahimi N Abdul S Mousavi M, W R P WORP. The study of relationship between optimism (positive thinking) and mental health (case study: students of Islamic Azad university of bandar abbas). Acad J Psychol Stud. (2017) 6:98–106.

[17] Schäfer SK Sopp MR Fuchs A Kotzur M Maahs L Michael T. The relationship between sense of coherence and mental health problems from childhood to young adulthood: a meta-analysis. J Affect Disord. (2023) 325:804–16. 10.1016/j.jad.2022.12.106 36638967

[18] Luthar SS Cicchetti D Becker B. The construct of resilience: a critical evaluation and guidelines for future work. Child Dev. (2000) 71(3):543–62. 10.1111/1467-8624.00164 10953923 PMC1885202

[19] Masten AS Barnes AJ. Resilience in children: developmental perspectives. Children. (2018) 5(7):98. 10.3390/children5070098 30018217 PMC6069421

[20] Bandura A. Self-efficacy: The exercise of control. Self-efficacy: The exercise of control. ix, 604–ix, 604 (1997).

[21] Rotter J. General expectancies for internal versus external control of reinforcement. Psychol Monogr. (1966) 80:1–28. 10.1037/h0092976 5340840

[22] Scheier M Carver C. Optimism, coping, and health: assessment and implications of generalized outcome expectancies. Health Psychol. (1985) 4:219–47. 10.1037//0278-6133.4.3.219 4029106

[23] Carver CS Scheier MF Segerstrom SC. Optimism. Clin Psychol Rev. (2010) 30(7):879–89. 10.1016/j.cpr.2010.01.006 20170998 PMC4161121

[24] Fairbank EJ Borenstein-Laurie J Alberts NM Wrosch C. Optimism, pessimism, and physical health among youth: a scoping review. J Pediatr Psychol. (2024) 49(8):580–95. 10.1093/jpepsy/jsae045 38879445 PMC11335150

[25] Herrman H Stewart DE Diaz-Granados N Berger EL Jackson B Yuen T. What is resilience? Can J Psychiatr. (2011) 56(5):258–65. 10.1177/070674371105600504 21586191

[26] Waddington J. Self-efficacy. ELT J. (2023) 77(2):237–40. 10.1093/elt/ccac046

[27] Galvin B Randel A Collins B Johnson R. Changing the focus of locus (of control): a targeted review of the locus of control literature and agenda for future research. J Organ Behav. (2018) 39:820–33. 10.1002/job.2275

[28] Branje S de Moor EL Spitzer J Becht AI. Dynamics of identity development in adolescence: a decade in review. J Res Adolesc. (2021) 31(4):908–27. 10.1111/jora.12678 34820948 PMC9298910

[29] Yoon S. Understanding family risk and protective factors that shape child development. Children. (2022) 9(9):1344. 10.3390/children9091344 36138653 PMC9497754

[30] Fleck L Fuchs A Moehler E Parzer P Koenig J Resch F Maternal bonding impairment predicts personality disorder features in adolescence: the moderating role of child temperament and sex. Personal Disord. (2021) 12(5):475–83. 10.1037/per0000433 33570973

[31] Moehler E Parzer P Brunner R Wiebel A Resch F. Emotional stress in pregnancy predicts human infant reactivity. Early Hum Dev. (2006) 82(11):731–7. 10.1016/j.earlhumdev.2006.02.010 16678983

[32] Moehler E Kagan J Parzer P Brunner R Reck C Wiebel A Childhood behavioral inhibition and maternal symptoms of depression. Psychopathology. (2007) 40(6):446–52. 10.1159/000107429 17709975

[33] Moehler E Brunner R Wiebel A Reck C Resch F. Maternal depressive symptoms in the postnatal period are associated with long-term impairment of mother–child bonding. Arch Womens Ment Health. (2006) 9(5):273–8. 10.1007/s00737-006-0149-5 16937313

[34] Joas J Hussong J Aktürk S Goth K Möhler E Honecker-Gebauer H. Maternal postnatal psychopathology predicts identity diffusion in young adult offspring. Children. (2024) 12(1):24. 10.3390/children12010024 39857855 PMC11763357

[35] Derogatis LR Lipman RS Covi L. SCL-90: an outpatient psychiatric rating scale–preliminary report. Psychopharmacol Bull. (1973) 9(1):13–28. 4682398

[36] Franke G. Eine weitere Überprüfung der symptom-check-liste (SCL-90-R) als forschungsinstrument. Diagnostica. (1992) 38:160–7.

[37] Borkenau P Ostendorf F. Untersuchungen zum fünf-faktoren-modell der persönlichkeit und seiner diagnostischen erfassung. Zeitschrift für Differentielle und Diagnostische Psychologie. (1989) 10(4):239–51.

[38] Costa P Mccrae R. Neo PI-R professional manual. Psychol Assess Res. (1992) 396.

[39] Richter V Guthke J. Leipziger Ereignis-und Belastungsinventar: (LEBI). Göttingen: Hogrefe, Verlag für Psychologie (1996). Available online at: https://books.google.de/books?id=ATnAngEACAAJ (Accessed September 09, 2026).

[40] Bergant A Nguyen T Moser R Ulmer H. Prävalenz depressiver Störungen im frühen Wochenbett. Gynäkol-geburtshilfliche Rundsch. (1999) 38(4):232–7. 10.1159/000022270 10325529

[41] Cox JL Holden JM Sagovsky R. Detection of postnatal depression: development of the 10-item Edinburgh postnatal depression scale. Br J Psychiatry. (1987) 150(6):782–6. 10.1192/bjp.150.6.782 3651732

[42] Brockington I Oates J George S Turner D Vostanis P Sullivan M A screening questionnaire for mother-infant bonding disorders. Arch Womens Ment Health. (2001) 3:133–40. 10.1007/s007370170010

[43] Kagan J Snidman N. Infant predictors of inhibited and uninhibited profiles. Psychol Sci. (1991) 2(1):40–4. 10.1111/j.1467-9280.1991.tb00094.x

[44] Kagan J. Galen’s Prophecy New York. New York: Basic Books[Google Scholar] (1994).

[45] Moehler E Kagan J Brunner R Wiebel A Kaufmann C Resch F. Association of behavioral inhibition with hair pigmentation in a European sample. Biol Psychol. (2006) 72(3):344–6. 10.1016/j.biopsycho.2005.12.001 16414174

[46] Perren S Groeben M Stadelmann S Klitzing K. Selbst-und fremdbezogene soziale Kompetenzen: Auswirkungen auf das emotionale Befinden (2008).

[47] Goth K Cloninger C Schmeck K. Das Junior Temperament and Charakter Inventar for das Kindergartenalter-JTCI/3-6. Klinik fur Psychhiatrie und Psychotherapie des Kindes-und Jugendalters der der JW Goethe-Universitat Frankfurt (2003).

[48] Hirshfeld-Becker DR Biederman J Henin A Faraone SV Davis S Harrington K Behavioral inhibition in preschool children at risk is a specific predictor of middle childhood social anxiety: a five-year follow-up. J Dev Behav Pediatr. (2007) 28(3):225–33. 10.1097/01.DBP.0000268559.34463.d0 (Accessed September 09, 2026). 17565290

[49] Zanarini MC Horwood J Wolke D Waylen A Fitzmaurice G Grant BF. Prevalence of DSM-IV borderline personality disorder in two community samples: 6,330 English 11-year-olds and 34,653 American adults. J Pers Disord. (2011) 25(5):607–19. 10.1521/pedi.2011.25.5.607 22023298 PMC4678770

[50] Derogatis LR. Brief symptom inventory. Eur J Psychol Assess. (1993).

[51] Moehler E Resch F. Behavioral inhibition at five years is predicted by a short questionnaire in infancy. Ment Health Addict Res. (2018) 3(4):1–6. 10.15761/MHAR.1000168

[52] Leppert K Koch B Brähler E Strauss B. Die resilienzskala (RS) – Überprüfung der Langfrom RS-25 und einer Kurzform RS-13. Klinische Diagnostik Eval. (2008) 1:226–43.

[53] Singer S Brähler E. Die» Sense of Coherence Scale «: Testhandbuch zur Deutschen Version. Parderborn: Vandenhoeck & Ruprecht (2007).

[54] Beierlein C Kemper C Kovaleva A Rammstedt B. Short scale for measuring general self-efficacy beliefs (ASKU). Methods, Data, Anal. (2013) 7(2):251–78. 10.12758/mda.2013.014

[55] Kemper C Beierlein C Kovaleva A Rammstedt B. Entwicklung und validierung einer ultrakurzen operationalisierung des konstrukts optimismus-pessimismus—die skala optimismus-pessimismus-2 (SOP2). Diagnostica. (2013) 59:119–29. 10.1026/0012-1924/a000089

[56] Kovaleva A Beierlein C Kemper CJ Rammstedt B. Eine Kurzskala zur Messung von Kontrollüberzeugung: Die Skala Internale-Externale-Kontrollüberzeugung-4 (IE-4) (2012). 10.1080/00223891.2013.836526 24066854

[57] Lohbeck A Schultheiß J Petermann F Petermann UJ. Die deutsche selbstbeurteilungs-version des strengths and difficulties questionnaire (SDQ-Deu-S). Diagnostica. (2015) 61:222–35. 10.1026/0012-1924/a000153

[58] Goodman R Renfrew D Mullick M. Predicting type of psychiatric disorder from strengths and difficulties questionnaire (SDQ) scores in child mental health clinics in London and Dhaka. Eur Child Adolesc Psychiatry. (2000) 9:129–34. 10.1007/s007870050008 10926063

[59] R Core Team. R: A Language and Environment for Statistical Computing. Vienna, Austria: R Foundation for Statistical Computing (2025). Available online at: https://www.R-project.org/ (Accessed September 09, 2026).

[60] Wickham H François R Henry L Müller K. dplyr: A grammar of data manipulation (2024). Available online at: https://CRAN.R-project.org/package=dplyr (Accessed September 09, 2026).

[61] Wickham H Vaughan D Girlich M. tidyr: Tidy messy data (2024) Available online at: https://CRAN.R-project.org/package=tidyr (Accessed September 09, 2026).

[62] Wickham H. Reshaping data with the reshape package (2007). Available online at: http://www.jstatsoft.org/v21/i12/ (Accessed September 09, 2026).

[63] John F Weisberg S. Companion to Applied Regression. Thousand Oaks (CA): Sage (2019). Available online at: https://www.john-fox.ca/Companion/ (Accessed September 09, 2026).

[64] Behrendt S. lm.beta: Add standardized regression coefficients to linear-model-objects (2023) Available online at: https://CRAN.R-project.org/package=lm.beta (Accessed September 09, 2026).

[65] Zeileis A Hothorn T. Diagnostic checking in regression relationships (2002). Available online at: https://CRAN.R-project.org/doc/Rnews/ (Accessed September 09, 2026).

[66] Revelle W. psych: Procedures for Psychological, Psychometric, and Personality Research. Illinois: Evanston (2025). Available online at: https://CRAN.R-project.org/package=psych (Accessed September 09, 2026).

[67] Bernaards CA Jennrich RI. Gradient projection algorithms and software for arbitrary rotation criteria in factor analysis. Educ Psychol Meas. (2005) 65(5):676–96. 10.1177/0013164404272507

[68] Harrell FJ. Hmisc: Harrell miscellaneous (2025). Available online at: https://CRAN.R-project.org/package=Hmisc (Accessed September 09, 2026).

[69] Wickham H. ggplot2: Elegant Graphics for Data Analysis. New York: Springer-Verlag (2016). Available online at: https://ggplot2.tidyverse.org (Accessed September 09, 2026).

[70] Wei T Simko V. R package “corrplot”: Visualization of a correlation matrix (2024). Available online at: https://github.com/taiyun/corrplot (Accessed September 09, 2026).

[71] Neuwirth E. RColorBrewer: ColorBrewer palettes (2022). Available online at: https://CRAN.R-project.org/package=RColorBrewer (Accessed September 09, 2026).

[72] Streiner D Norman G Cairney J. Health measurement scales: a practical guide to their development and use (5th edition). Aust N Z J Public Health. (2016) 40(3):294–5. 10.1111/1753-6405.12484 27242256

[73] Benjamini Y Hochberg Y. Controlling the false discovery rate: a practical and powerful approach to multiple testing. J R Stat Soc Ser B Methodol. (1995) 57(1):289–300. 10.1111/j.2517-6161.1995.tb02031.x

[74] Kline RB. Principles and Practice of Structural Equation Modeling. 4th ed. New York: Methodology in the Social Sciences (2016). p. xvii, 534.

[75] Kenny DA Kaniskan B McCoach DB. The performance of RMSEA in models with small degrees of freedom. Sociol Methods Res. (2015) 44(3):486–507. 10.1177/0049124114543236

[76] Schäfer SK Fritz J Sopp MR Kunzler AM von Boros L Tüscher O Interrelations of resilience factors and their incremental impact for mental health: insights from network modeling using a prospective study across seven timepoints. Transl Psychiatry. (2023) 13(1):328. 10.1038/s41398-023-02603-2 37872216 PMC10593776

[77] Dawel A Shou Y Gulliver A Cherbuin N Banfield M Murray K Cause or symptom? A longitudinal test of bidirectional relationships between emotion regulation strategies and mental health symptoms. Emotion. (2021) 21:1511–21. 10.1037/emo0001018 34843310

[78] Lamers S Westerhof G Glas C Bohlmeijer E. The bidirectional relation between positive mental health and psychopathology in a longitudinal representative panel study. J Posit Psychol. (2015) 10:553–60. 10.1080/17439760.2015.1015156

[79] Zimmer-Gembeck MJ Skinner EA. The development of coping: implications for psychopathology and resilience. In: Cicchetti D, editor. Developmental Psychopathology: Risk, Resilience, and Intervention. 3rd ed. John Wiley & Sons, Inc (2016). p. 485–545. Available online at: https://onlinelibrary.wiley.com/doi/abs/10.1002/9781119125556.devpsy410 (Accessed September 09, 2026).

[80] Paersch C Recher D Schulz A Henninger M Schlup B Künzler F Self-efficacy effects on symptom experiences in daily life and early treatment success in anxiety patients. Clin Psychol Sci. (2025) 13(1):178–94. 10.1177/21677026231205262 39831174 PMC11735308

[81] Posadzki P Stockl A Musonda P Tsouroufli M. A mixed-method approach to sense of coherence, health behaviors, self-efficacy and optimism: towards the operationalization of positive health attitudes. Scand J Psychol. (2010) 51:246–52. 10.1111/j.1467-9450.2009.00764.x 20132461

[82] Reis J Nunes F Matos PM Mota CP. Are attachment to parents and self-efficacy linked with emerging adults’ values of future expectations? Br J Dev Psychol. (2025) 43(3):742–54. 10.1111/bjdp.12548 39895185

[83] Goodman SH Rouse MH Connell AM Broth MR Hall CM Heyward D. Maternal depression and child psychopathology: a meta-analytic review. Clin Child Fam Psychol Rev. (2011) 14(1):1–27. 10.1007/s10567-010-0080-1 21052833

[84] Plant DT Pawlby S Pariante CM Jones FW. When one childhood meets another—maternal childhood trauma and offspring child psychopathology: a systematic review. Clin Child Psychol Psychiatry. (2018) 23(3):483–500. 10.1177/1359104517742186 29171287

[85] Suveg C Shaffer A Morelen D Thomassin K. Links between maternal and child psychopathology symptoms: mediation through child emotion regulation and moderation through maternal behavior. Child Psychiatr Hum Dev. (2011) 42(5):507–20. 10.1007/s10578-011-0223-8 21484417

[86] Barnes J Theule J. Maternal depression and infant attachment security: a meta-analysis. Infant Ment Health J. (2019) 40(6):817–34. 10.1002/imhj.21812 31415711

[87] Maughan A Cicchetti D Toth SL Rogosch FA. Early-occurring maternal depression and maternal negativity in predicting young children’s emotion regulation and socioemotional difficulties. J Abnorm Child Psychol. (2007) 35(5):685–703. 10.1007/s10802-007-9129-0 17503175

[88] Cicchetti D Blender JA. A multiple-levels-of-analysis perspective on resilience. Ann N Y Acad Sci. (2006) 1094(1):248–58. 10.1196/annals.1376.029 17347356

[89] Parsaee S Jahani N Salehiniya H Raeison M. Effectiveness of mindfulness training in emotion regulation and distress tolerance among breast cancer patients. J Breast Dis. (2024) 17:84–101. 10.61186/ijbd.17.1.84

[90] Salmabadi M Rajabi MJ Safara M. Effectiveness of training the review of life on life satisfaction and sense of coherence middle-aged women and elderly nursing home residents in Qazvin. Iran J Ageing. (2018) 13(2):198 EP–209. 10.32598/sija.13.2.198

[91] Sayahi HS Vakili SV Asaseh MA. The effectiveness of role-playing training on emotional self-disclosure and sense of coherence in students with hearing loss. Educ Res Med Sci (2024) 13(1):e149418. 10.5812/ermsj-149418

[92] Tan YR Ooi YP Ang RP Goh DH Kwan C Fung DS Feasibility trial of virtual reality exposure therapy for selective mutism. Clin Child Psychol Psychiatry. (2022) 27(2):351–68. 10.1177/13591045211056920 34866415

[93] Vastamäki J Moser K Paul KI. How stable is sense of coherence? Changes following an intervention for unemployed individuals. Scand J Psychol. (2009) 50(2):161–71. 10.1111/j.1467-9450.2008.00695.x 18980600

[94] Goldbach N Reif A Preuss H Röhm M Straus E Streicher E The role of resources in the face of psychopathology. J Clin Psychol. (2020) 76(3):406–22. 10.1002/jclp.22884 31777087

[95] Taris TW Kompier MAJ. Cause and effect: optimizing the designs of longitudinal studies in occupational health psychology. Work Stress. (2014) 28(1):1–8. 10.1080/02678373.2014.878494

[96] van de Mortel TF. Faking it: social desirability response bias in self-report research. Aust J Adv Nurs. (2008) 25(4):40–8.

[97] Henrich J Heine SJ Norenzayan A. The weirdest people in the world? Behav Brain Sci. (2010) 33(2–3):61–83. 10.1017/S0140525X0999152X 20550733

[98] De Los Reyes A Augenstein T Wang M Thomas S Drabick D Burgers D The validity of the multi-informant approach to assessing child and adolescent mental health. Psychol Bull. (2015) 141:858–900. 10.1037/a0038498 25915035 PMC4486608

