# Supplementary material for: Tracing psychological resources and mental health: the role of sense of coherence, resilience and self-efficacy in young adulthood
Source: Front Child Adolesc Psychiatry. 2026 Apr 30;5:1759711. doi: 10.3389/frcha.2026.1759711 (PMC13171495; doi:10.3389/frcha.2026.1759711)
Supplement: Supplementary file 1 [file Table1.docx]

**Table S1**

*Descriptives and correlations*

| **Variable** | ***M* (*SD*)** | **1.** | **2.** | **3.** | **4.** | **5.** | **6.** | **7.** | **8.** | **9.** | **10.** | **11.** | **12.** | **13.** | **14.** | **15.** | **16.** | **17.** | **18.** |
| --- | --- | --- | --- | --- | --- | --- | --- | --- | --- | --- | --- | --- | --- | --- | --- | --- | --- | --- | --- |
| 1. Adolescent sex | – |  |  |  |  |  |  |  |  |  |  |  |  |  |  |  |  |  |  |
| 2. Adolescent psychopathology T7 | 10.14 (5.28) | 0.18 |  |  |  |  |  |  |  |  |  |  |  |  |  |  |  |  |  |
| 3. Adolescent psychopathology T6 | 9.25 (5.24) | 0.21 | 0.57*** |  |  |  |  |  |  |  |  |  |  |  |  |  |  |  |  |
| 4. Adolescent resilience T7 | 69.49 (11.02) | 0.09 | –0.58*** | –0.42** |  |  |  |  |  |  |  |  |  |  |  |  |  |  |  |
| 5. Adolescent SOC T7 | 44.92 (9.85) | 0.08 | –0.77*** | –0.48*** | 0.74*** |  |  |  |  |  |  |  |  |  |  |  |  |  |  |
| 6. Adolescent optimism T7 | 5.14 (1.28) | 0.10 | –0.52*** | –0.30* | 0.42** | 0.66*** |  |  |  |  |  |  |  |  |  |  |  |  |  |
| 7. Adolescent LOC internal T7 | 4.11 (0.63) | –0.01 | –0.38** | –0.33* | 0.45*** | 0.48*** | 0.26 |  |  |  |  |  |  |  |  |  |  |  |  |
| 8. Adolescent LOC external T7 | 2.16 (0.75) | –0.06 | 0.31* | 0.11 | –0.20 | –0.31* | –0.19 | –0.31* |  |  |  |  |  |  |  |  |  |  |  |
| 9. Adolescent self-efficacy T7 | 3.99 (0.56) | -0.09 | –0.48*** | –0.30* | 0.68*** | 0.51*** | 0.40** | 0.58*** | –0.31* |  |  |  |  |  |  |  |  |  |  |
| 10. Adolescent SC self-oriented T7 | 15.15 (3.57) | –0.04 | –0.03 | 0.03 | 0.09 | 0.00 | –0.05 | 0.12 | –0.21 | 0.12 |  |  |  |  |  |  |  |  |  |
| 11. Adolescent SC other-oriented T7 | 16.36 (2.71) | 0.22 | –0.16 | –0.11 | 0.23 | 0.26 | 0.26 | 0.00 | –0.20 | –0.10 | 0.26 |  |  |  |  |  |  |  |  |
| 12. Adolescent SC peer-relations T7 | 9.32 (1.11) | –0.03 | –0.25 | –0.15 | 0.14 | 0.07 | –0.14 | 0.12 | –0.08 | 0.08 | 0.35* | 0.08 |  |  |  |  |  |  |  |
| 13. Bonding impairment (T1-T4) | 17.16 (2.82) | –0.02 | 0.20 | –0.02 | –0.27 | –0.27 | –0.20 | –0.27 | 0.23 | –0.31* | –0.02 | 0.02 | –0.07 |  |  |  |  |  |  |
| 14. Bonding rejection anger (T1-T4) | 8.80 (1.58) | 0.18 | 0.21 | 0.12 | –0.15 | –0.13 | –0.19 | –0.20 | 0.09 | –0.29* | –0.15 | –0.08 | –0.14 | 0.55*** |  |  |  |  |  |
| 15. Bonding fear care (T1-T4) | 6.13 (1.06) | –0.03 | 0.01 | –0.12 | –0.13 | –0.06 | 0.01 | –0.16 | 0.12 | –0.07 | –0.16 | –0.20 | –0.16 | 0.57*** | 0.43** |  |  |  |  |
| 16. Bonding risk maltreatment (T1-T4) | 2.13 (0.31) | 0.00 | 0.04 | –0.06 | –0.24 | –0.06 | 0.01 | –0.14 | 0.30* | –0.35* | –0.11 | 0.19 | –0.08 | 0.35* | 0.13 | 0.04 |  |  |  |
| 17. Maternal education T1 | – | 0.00 | –0.36** | –0.18 | 0.16 | 0.29* | 0.21 | 0.24 | –0.25 | 0.24 | 0.09 | 0.18 | 0.08 | 0.00 | –0.09 | 0.01 | 0.36** |  |  |
| 18. Maternal psychopathology (T1-T5) | 0.23 (0.15) | –0.11 | 0.18 | 0.08 | –0.25 | –0.22 | –0.13 | –0.14 | 0.34* | –0.15 | -0.17 | –0.15 | –0.30* | 0.30* | 0.27 | 0.20 | 0.28* | –0.05 |  |

*Note.* Spearman rank-order correlations are reported. Significance levels: **p* < .05, ***p* < .01, ****p* < .001. Sex was contrast coded (–1 = male, 1 = female). LOC = Locus of control. SC = social competences.
